# Functional Characterization of Human Cancer-Derived TRKB Mutations

**DOI:** 10.1371/journal.pone.0016871

**Published:** 2011-02-17

**Authors:** Thomas R. Geiger, Ji-Ying Song, Aranzazu Rosado, Daniel S. Peeper

**Affiliations:** 1 Division of Molecular Genetics, Netherlands Cancer Institute, Amsterdam, the Netherlands; 2 Department of Experimental Animal Pathology, Netherlands Cancer Institute, Amsterdam, the Netherlands; Emory University, United States of Ameica

## Abstract

Cancer originates from cells that have acquired mutations in genes critical for controlling cell proliferation, survival and differentiation. Often, tumors continue to depend on these so-called driver mutations, providing the rationale for targeted anticancer therapies. To date, large-scale sequencing analyses have revealed hundreds of mutations in human tumors. However, without their functional validation it remains unclear which mutations correspond to driver, or rather bystander, mutations and, therefore, whether the mutated gene represents a target for therapeutic intervention. In human colorectal tumors, the neurotrophic receptor TRKB has been found mutated on two different sites in its kinase domain (TRKB^T695I^ and TRKB^D751N^). Another site, in the extracellular part of TRKB, is mutated in a human lung adenocarcinoma cell line (TRKB^L138F^). Lastly, our own analysis has identified one additional TRKB point mutation proximal to the kinase domain (TRKB^P507L^) in a human melanoma cell line. The functional consequences of all these point mutations, however, have so far remained elusive. Previously, we have shown that TRKB is a potent suppressor of anoikis and that TRKB-expressing cells form highly invasive and metastatic tumors in nude mice. To assess the functional consequences of these four TRKB mutations, we determined their potential to suppress anoikis and to form tumors in nude mice. Unexpectedly, both colon cancer-derived mutants, TRKB^T695I^ and TRKB^D751N^, displayed reduced activity compared to that of wild-type TRKB. Consistently, upon stimulation with the TRKB ligand BDNF, these mutants were impaired in activating TRKB and its downstream effectors AKT and ERK. The two mutants derived from human tumor cell lines (TRKB^L138F^ and TRKB^P507L^) were functionally indistinguishable from wild-type TRKB in both *in-vitro* and *in-vivo* assays. In conclusion, we fail to detect any gain-of-function of four cancer-derived TRKB point mutations.

## Introduction

Cancer is a genetic disease, with numerous somatic mutations in proto-oncogenes and tumor suppressor genes contributing to the malignant phenotype [1]. These genes normally control vital processes including cell proliferation, survival or differentiation [2]. Their mutation endows tumor cells with a selective advantage, resulting in clonal expansion and neoplasia. Although tumors usually harbor multiple genetic aberrations [1], inhibition of only one or a few gene products can be sufficient to largely suppress tumor cell proliferation or viability [3]. This has been shown to cause tumor regression in various cancer mouse models [4]–[6] and led to the concepts of “oncogene addiction” and “tumor suppressor gene hypersensitivity” [3]. The dependency of tumor cells on certain oncogenes and signaling pathways exposes an “Achilles' heel” of cancer, which can be targeted for anticancer therapy [7]. Based on this concept, several novel therapeutics have been developed and are used in the clinic [8]. They include imatinib mesylate (or “Gleevec”/“Glivec”) for BCR-ABL inhibition in Chronic Myeloid Leukemia (CML) [9] and for KIT inhibition in gastrointestinal stromal tumors (GIST) [10], respectively. Similarly, in breast cancer patients with ERBB2 (also named HER-2/NEU) overexpression, the monoclonal antibody trastuzumab [11]–[13] and the small molecule inhibitor lapatinib [14] are effective. A critical role for oncogenic mutations is illustrated by the example of epidermal growth factor receptor (EGFR/ERBB1) in Non Small Cell Lung Cancer (NSCLC), where only a subset of patients respond to the EGFR inhibitor gefitinib. Sequencing analyses revealed that responsive tumors harbor specific mutations in EGFR, increasing its activation by EGF [15].

These and other examples illustrate that the identification of oncogenes critically required for tumor cell proliferation and survival can lead to effective anticancer therapeutics. Therefore, several research groups have been carrying out systematic large-scale sequencing analyses to screen for genes that are mutated in cancer. This strategy has led first to the identification of the BRAF kinase as a critical oncogene in a large proportion of melanomas and several other cancers [16]. In 2003, the group of Vogelstein, Kinzler and Velculescu systematically sequenced the kinase domains of all tyrosine kinases in a collection of human colorectal cancers. They found 7 out of 138 genes analyzed to be mutated in more than one tumor [17]. The same group subsequently analyzed more than 1000 different genes in breast and colon cancer [18], identifying up to 189 novel and known candidate cancer genes. Likewise, Stratton, Futreal and co-workers at the Sanger Institute sequenced first 518 full-length kinases in lung tumors and tumor cell lines [19] and subsequently the full kinome in ten different cancer types [20]. These and other analyses have identified hundreds of novel somatic mutations across several human malignancies [21]. However, a few exceptions aside, the functional consequences of those mutations have remained largely elusive. To select the appropriate targets for future anticancer therapies, it will be necessary to test experimentally which of these mutations are oncogenic and on which mutated genes tumors depend.

We chose to investigate the neurotrophic receptor TRKB (NTRK2), for which three somatic, non-synonymous point mutations have been reported in the studies mentioned above [17], [19], while our own analysis has revealed another TRKB point mutation in a human melanoma cell line. TRKB is one of three members of the TRK receptor family, which also includes TRKA and TRKC. TRKB preferentially binds the neurotrophin brain-derived neurotrophic factor (BDNF) and NT4/5, while TRKA and TRKC have high affinities to nerve growth factor (NGF) and NT3, respectively [22]. The pan-TRK receptor p75NTR further modulates the responsiveness of TRK receptors to neurotrophins [22]. TRKB has been found overexpressed in a number of aggressive human cancer types (for review see [23]). Previously, we have shown that TRKB is a potent suppressor of anoikis (apoptosis induced by inappropriate or absent cell adhesion) in rat epithelial cells, and that TRKB-overexpressing cells form highly invasive and metastatic tumors in nude mice [24]. Furthermore, our previously reported structure-function analysis demonstrated that all these functions depend on TRKB kinase activity in this experimental system [25]. More recently, we have demonstrated for TRKB-transformed rat epithelial cells that increased MAPK activity signals via twist and snail to induce Epithelial-Mesenchymal Transition (EMT)-like transformation, anoikis suppression and metastasis [26]. As two of the identified cancer-derived TRKB point mutations map within the kinase domain, possibly affecting its enzymatic activity, it is conceivable that they act oncogenically and correspond to driver mutations. The aim of this study was, therefore, to assess the functional consequences of four independent human cancer-derived TRKB point mutations.

## Results

### Identification of human cancer-derived TRKB point mutants and generation of cell systems

**Figure 1:**
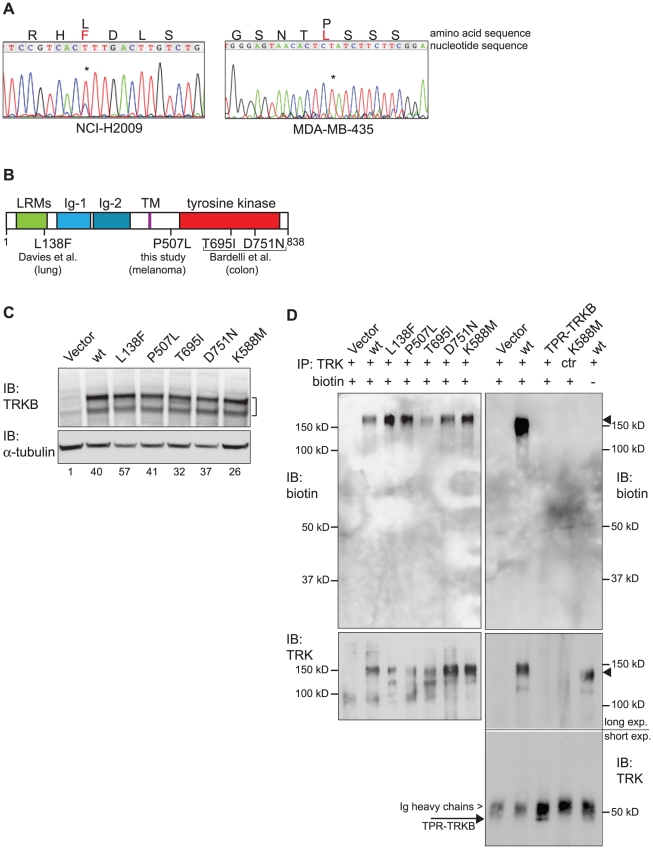
Identification of human cancer-derived TRKB point mutants and generation of cell systems. (**A**) Sequencing analysis of gDNA from NCI-H2009 cells harboring the TRKB^L138F^ mutation (left panel) and from MDA-MB-435 cells harboring the TRKB^P507L^ mutation (right panel). Asterisks (*) indicate the respective mutated bases. Black letters denote wild-type residues, red letters denote mutant residues. (**B**) Schematic overview over all cancer-derived TRKB point mutations analyzed in this study. LRM: Leucine-Rich Motif, Ig: Immunoglobulin-like domain, TM: transmembrane domain. Numbers indicate positions of amino acid residues. (**C**) Expression levels of mutant or wild-type TRKB in RIE-1 cells, analyzed on immunoblot (IB). Tubulin serves as loading control. Numbers represent quantification of TRKB signal, normalized to alpha-tubulin, and relative to Vector control. (**D**) Cell surface biotinylation assay showing that at least a significant fraction of all TRKB mutants localizes to the cell membrane. Total cell surface proteins were biotinylated with Sulfo-NHS-LC-Biotin, lysed and TRKB was immunoprecipitated (IP) with TRK antibody (C-14, C-13 for control). After gel electrophoresis, biotinylated TRKB was visualized with streptavidin-HRP and total TRKB with TRK antibody (C-14). All wild-type and mutant TRKB proteins became biotinylated (upper left panel). On the right hand side the specificity of the assay is demonstrated: biotin signal was only detected for full-length TRKB (upper right panel, second lane), but not for cytosolic, truncated TPR-TRKB [25] (third lane, expected at ∼50 kD), and not in the control IP (lane 4) or in the absence of Sulfo-NHS-LC-Biotin (lane 5). IP of total full-length and truncated TRKB is shown in bottom panels. Arrowheads indicate full-length TRKB, arrow indicates truncated TPR-TRKB (just below Ig heavy chains).

Two non-synonymous point mutations within the kinase domain of the *TRKB* gene have been discovered in colorectal tumors: TRKB^T695I^ and TRKB^D751N^ [17] (the numbering of all amino acid sequences refers to the full length human TRKB protein, accession number NP_006171.2). Another TRKB point mutation, TRKB^L138F^, was identified in the lung adenocarcinoma cell line NCI-H2009 [19]. This mutation lies within the leucine-rich domain of the extracellular part of the receptor, which has been shown to be required for binding of the TRKB ligand brain-derived neurotrophic factor (BDNF) and activation of the TRKB receptor [25], [27], [28]. We confirmed the presence of this mutation by sequencing genomic DNA (gDNA) isolated from NCI-H2009 cells (Figure 1A, left panel). The presence of a double peak (thymidine and cytosine) indicates that the mutation is heterozygous, consistent with the original report [19]. It results in the substitution of leucine by phenylalanine. To search for more cancer-associated *TRKB* point mutations, we sequenced the exons comprising the *TRKB* kinase domain from genomic DNA of 28 human tumor cell lines, from several tissue origins (data not shown). This analysis revealed one additional *TRKB* point mutation, in the MDA-MB-435 melanoma cell line. (The MDA-MB-435 cell line seems to have been classified wrongly in the past [29]. Whereas it was originally isolated from a patient with breast cancer [30], [31], recent analysis indicated that the currently available cell line is of melanoma origin [32], [33]). The *TRKB* mutation identified in MDA-MB-435 cells is C1520T, resulting in a substitution of proline 507 with leucine (TRKB^P507L^). In this case, the sequencing of gDNA revealed a single peak only (Figure 1A right panel), suggesting that the mutation is homozygous. The P507 residue is located proximal to the kinase domain in the intracellular part of the receptor. Figure 1B shows a schematic overview of the positions of all human cancer-derived TRKB point mutations analyzed in this study.

We assessed whether these four cancer-derived TRKB point mutations correspond to gain-of-function mutations associated with increased oncogenic potential. We performed this analysis in rat epithelial cells first, because they are highly sensitive to TRKB-mediated oncogenic transformation, as marked by an EMT-like morphologic transformation, anoikis resistance and metastatic tumor formation in nude mice [24]–[26]. To this end, we cloned wild-type and mutant TRKB into the retroviral pBabe-puro expression vector and transduced rat intestinal epithelial (RIE-1) cells as well as E1A-immortalized rat kidney epithelial (RK3E) cells. Both cell lines are immortalized, yet non-oncogenic in athymic mice. As a negative control, we expressed a kinase-inactive mutant of TRKB, TRKB^K588M^ [34], which we previously demonstrated to be incapable of oncogenically transforming RIE-1 cells [25], [26]. We confirmed by western blot analysis that all TRKB mutants were expressed to similar levels as wild-type TRKB (Figure 1C for RIE-1 cells, Figure S1A for RK3E cells). Consistent with our previous observations [25], we detected TRKB as multiple species on an SDS-polyacrylamide gel, most likely reflecting differentially glycosylated species [35]. Furthermore, by performing a cell surface biotinylation assay we ensured that at least a detectable fraction of all the TRKB mutants properly localized to the cell membrane [36] (Figure 1D). We thus generated a cell system allowing us to compare the activities and functions of the different TRKB mutants side by side.

### Transforming potential of human cancer-derived TRKB point mutants in vitro

**Figure 2:**
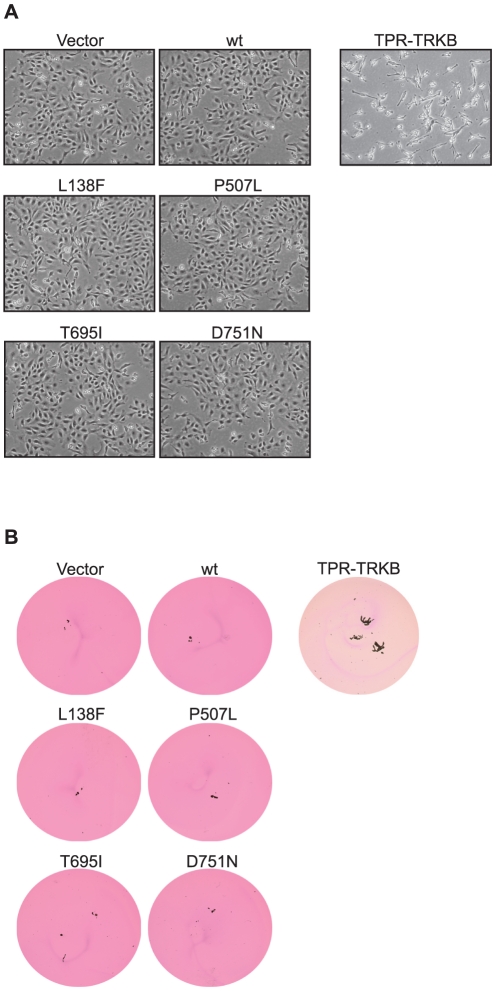
Transforming potential of human cancer-derived TRKB point mutants *in vitro*. (**A**) Morphology of RIE-1 cells expressing mutant or wild-type TRKB, photographed at 50x magnification. (**B**) Anoikis assay, in which cells described in (A) were seeded onto ULC plates and scanned at 1x magnification 9 days later (7 days for TPR-TRKB).

Like most kinases, TRKB needs a minimal level of activation to transform epithelial cells. We hypothesized that if the cancer-derived TRKB mutations confer a gain of function, they may transform epithelial cells even in the absence (or with reduced levels) of BDNF. However, when we tested the various TRKB mutants in the absence of co-expressed ligand, none of them induced a spindle-shaped cell morphology (Figure 2A for RIE-1 cells, Figure S1B for RK3E cells) or suppressed anoikis (Figure 2B, Figure S1C) in the absence of BDNF. This was in contrast to what was observed for the constitutively active and ligand-independent TPR-TRKB mutant, which did transform RIE-1 and RK3E cells and suppressed anoikis in the absence of exogenous BDNF, consistent with our previous report [25].

**Figure 3:**
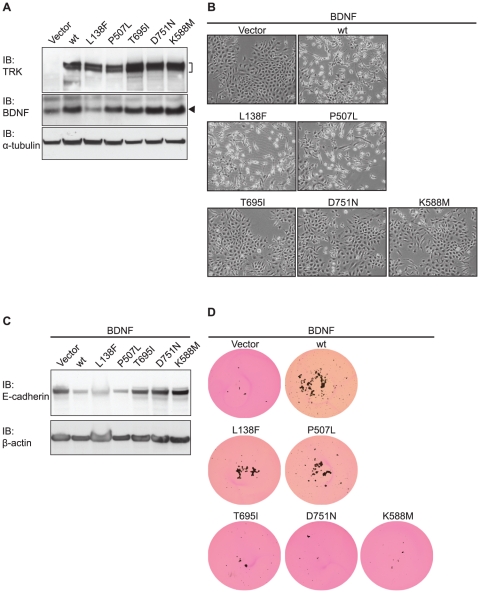
Transforming potential of BDNF-activated TRKB point mutants *in vitro*. (**A**) RIE-1 cells expressing mutant or wild-type TRKB were transduced with BDNF and analyzed on immunoblot (IB). Arrowhead indicates position of BDNF. Tubulin serves as loading control. (**B**) Morphologic transformation of RIE-1 cells co-expressing mutant or wild-type TRKB and BDNF, photographed at 50x magnification. (**C**) The epithelial marker E-cadherin is downregulated in TRKB-induced, morphologically transformed, cells, as assessed by immunoblot (IB) analysis. β-actin serves as loading control. (**D**) Anoikis suppression by mutant or wild-type TRKB+BDNF in cells described in (A). Cells were scanned at 1x magnification 9 days after seeding onto ULC plates.

Next, we activated the wild-type and mutant receptors by stably co-expressing human BDNF, both in RIE-1 (Figure 3A) and RK3E cells (Figure S1D). Consistent with our previous observations [24]–[26], for cells expressing wild-type TRKB+BDNF this resulted in EMT-like morphologic transformation (Figure 3B, Figure S1E), downregulation of E-cadherin (Figure 3C and Figure S1F) and anoikis suppression (Figure 3D, Figure S1G). The same was seen for cells expressing TRKB^L138F^+BDNF and TRKB^P507L^+BDNF. By contrast, TRKB^T695I^– and TRKB^D751N^–expressing cells were impaired in their response to BDNF (Figure 3B,C,D, Figure S1E,F,G). These results show that, unexpectedly, TRKB^T695I^ and TRKB^D751N^ are impaired in their ability to transform rat epithelial cells *in vitro*. Furthermore, TRKB^L138F^ and TRKB^P507L^ are indistinguishable from wild-type TRKB in this setting.

### Responsiveness of human cancer-derived TRKB mutants to BDNF

**Figure 4:**
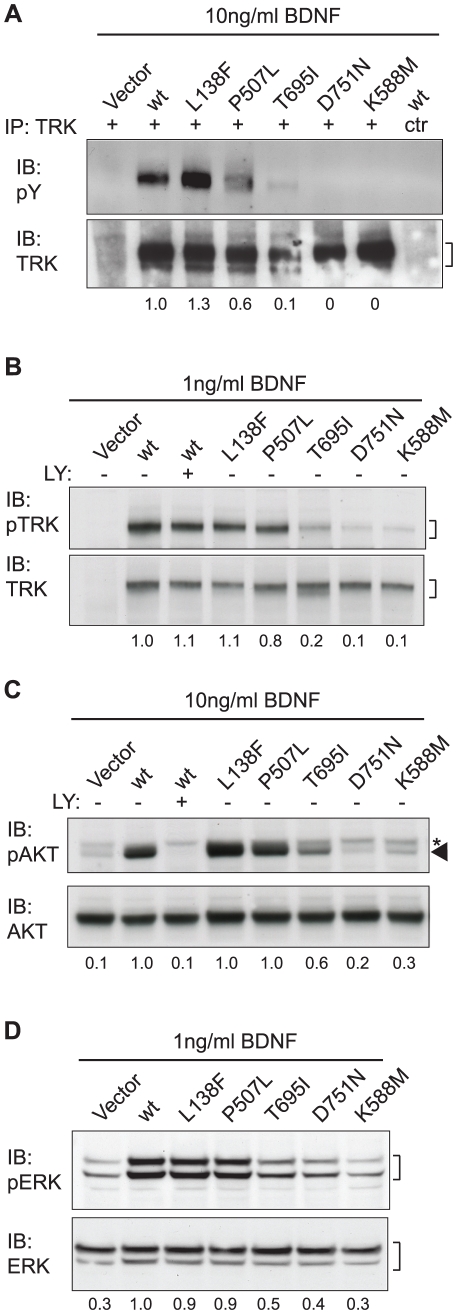
Responsiveness of human cancer-derived TRKB mutants to BDNF. (**A**) RIE-1 cells expressing wild-type or mutant TRKB were stimulated with 10 ng/ml recombinant BDNF and analyzed for phospho-tyrosine (pY) and total TRK content by immunoprecipitation (IP) and subsequent immunoblot (IB) analysis. (**B**) RIE-1 cells expressing wild-type or mutant TRKB were stimulated with 1 ng/ml recombinant BDNF and analyzed for phospho (p) and total TRK. (**C**) RIE-1 cells expressing wild-type or mutant TRKB were stimulated with 10 ng/ml recombinant BDNF and analyzed for phospho- (p) AKT and total AKT. PI3K inhibitor LY294002 was applied to confirm the identity of the pAKT signal indicated by the arrowhead. Asterisk (*) indicates a non-specific band. The samples loaded in lanes two and three serve as controls and were derived from a replicate experiment performed under identical conditions. (D) RIE-1 cells expressing wild-type or mutant TRKB were stimulated with 1 ng/ml recombinant BDNF and analyzed for phospho- (p) ERK and total ERK. For all panels, the numbers underneath indicate quantification of the phospho-specific signals, normalized to the total signals and relative to those of wild-type TRKB.

We have previously shown that TRKB-mediated oncogenic transformation of RIE-1 cells critically depends on TRKB kinase activity [25], [26]. In search of a biochemical explanation for the unanticipated results described above, we determined whether the cancer-derived TRKB mutants differ from wild-type TRKB in their responsiveness to BDNF. To measure TRKB activation, we used RIE-1 cells expressing wild-type or mutant TRKB but no ligand, and stimulated the cells with a physiologically relevant range of recombinant BDNF. In line with our previous studies [25], [26], this induced autophosphorylation of wild-type TRKB (Figure 4A and 4B), and led to the activation of two major downstream signaling pathways [22]: the PI3K pathway (resulting in phosphorylation of AKT/PKB; Figure 4C) and the MAPK pathway (resulting in phosphorylation of MAPK/ERK; Figure 4D). Exposure to 1 ng/ml BDNF was sufficient to elicit wild-type TRKB autophosphorylation and activate the MAPK pathway, whereas higher concentrations of BDNF were required to activate AKT (Figure 4B,C,D and data not shown). TRKB^L138F^ and TRKB^P507L^ responded to BDNF similarly to wild-type TRKB. Consistent with their inability to suppress anoikis, TRKB^T695I^ was only partially activated by BDNF, while TRKB^D751N^ was completely unresponsive to BDNF, identical to kinase-inactive TRKB^K588M^ (Figure 4). These results show that TRKB^T695I^ and TRKB^D751N^ display reduced responsiveness to BDNF stimulation in rat epithelial cells, whereas TRKB^L138F^ and TRKB^P507L^ behave indistinguishably from wild-type TRKB.

### Oncogenic potential of human cancer-derived TRKB mutants expressed in rat epithelial cells

**Figure 5:**
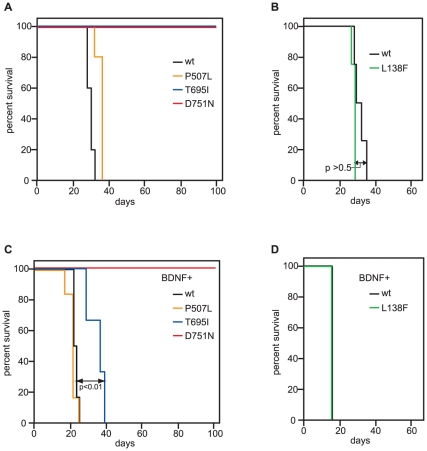
Oncogenic potential of human cancer-derived TRKB mutants expressed in RIE-1 cells. (**A**) 1*10^6^ RIE-1 cells expressing TRKB (wild-type or mutant) were subcutaneously injected into both flanks of nude mice. n = 5 for wt and P507L, n = 4 for T695I and D751N. (**B**) 1*10^6^ RIE-1 cells expressing TRKB (wild-type or mutant) were subcutaneously injected into both flanks of nude mice. n = 4 for both cell lines. (**C**) 1*10^4^ RIE-1 cells co-expressing BDNF and TRKB (wild-type or mutant) were subcutaneously injected into nude mice. n = 6 for wt and P507L, n = 3 for T695I and D751N. (**D**) 1*10^5^ RIE-1 cells co-expressing BDNF and TRKB (wild-type or mutant) were subcutaneously injected into nude mice. n = 4 for both cell lines. In all experiments, mice were sacrificed when tumor burden reached 2 cm^2^ and Kaplan-Meier survival curve is shown. Statistical significance was determined with a Log-Rank test.

Next, we tested the oncogenic potential of the TRKB mutants *in vivo*, in mouse xenograft experiments. We subcutaneously inoculated Balb/c nude mice with wild-type or mutant TRKB-expressing cells. In the absence of BDNF, only wild-type TRKB-, TRKB^L138F^- and TRKB^P507L^-expressing RIE-1 cells (Figure 5A,B, Figure S2A,B) and RK3E cells (Figure S2E,F) formed large tumors with short latencies, but none of the TRKB^T695I^- or TRKB^D751N^-expressing cells did. Similar to this, also in the presence of co-expressed BDNF, TRKB^L138F^ and TRKB^P507L^ caused tumors similarly to wild-type TRKB in RIE-1 and RK3E cells (Figure 5C,D, Figure S2C,D and Figure S2G,H). In this setting, RIE-1 TRKB^T695I^+BDNF–expressing cells also formed tumors, but with a statistically significant delay, compared to RIE-1 TRKB^wt^+BDNF–expressing cells (Figure 5C, S2C). Of note, when 1*10^5^ cells were inoculated, also RIE-1 TRKB^D751N^+BDNF-expressing cells formed tumors, but with a significant longer latency than those induced by RIE-1 TRKB^wt^+BDNF–expressing cells (data not shown). In RK3E cells, expression of TRKB^D751N^ did not lead to tumor formation, even with high cell numbers and in the presence of BDNF, whereas RK3E TRKB^T695I^+BDNF-expressing cells formed tumors with a significant longer latency compared to RK3E TRKB^wt^+BDNF-expressing cells (Figure S2G). Notwithstanding some differences between the two cell lines, overall, these findings indicate that TRKB^T695I^ and TRKB^D751N^ are impaired in transforming rat epithelial cells also *in vivo*. Furthermore, neither TRKB^L138F^ nor TRKB^P507L^ displays increased oncogenic activity compared to wild-type TRKB.

### BDNF responsiveness of human colon cancer-derived TRKB^T695I^ and TRKB^D751N^ expressed in colon cancer cells

**Figure 6:**
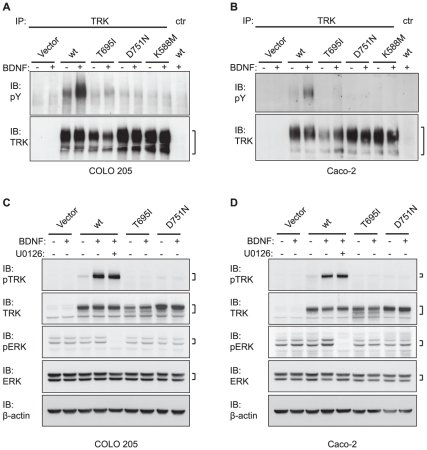
BDNF responsiveness of human colon cancer-derived TRKB^T695I^ and TRKB^D751N^ expressed in colon cancer cells. (**A**) COLO 205 cells and (**B**) Caco-2 cells expressing wild-type or mutant TRKB were stimulated with 10 ng/ml recombinant BDNF and analyzed for phospho-tyrosine (pY) content by immunoprecipitation (IP) and subsequent immunoblot (IB) analysis. (**C**) Cell lysates of COLO 205 cells from (A) and, (**D**) lysates of Caco-2 cells from (B) were analyzed for phospho (p) and total TRK and (p)ERK. The MEK inhibitor U0126 was applied to confirm the identity of the pERK signals. β-actin serves as loading control.

One possible explanation for the impaired activity of TRKB^T695I^ and TRKB^D751N^ in the assays described above is that we performed them in an aphysiological cellular context. Indeed, the wiring of the intracellular signaling networks and the expression pattern of TRKB regulatory factors may not be identical across cell types. Ideally, one should assess the function of the TRKB mutants in their original context, that is, in the tumor cells in which they were originally identified. However, there are no cell lines available that express TRKB^T695I^ or TRKB^D751N^ endogenously. Moreover, to our knowledge there is no primary human colon epithelial cell line available. Aiming to use a physiologically relevant cell system, we transduced COLO 205 and Caco-2 human colon carcinoma cells with TRKB^T695I^ or TRKB^D751N^ and obtained polyclonal populations stably expressing either receptor. We stimulated these cells with recombinant BDNF and subsequently measured TRKB autophosphorylation and activation of downstream effectors. Similar to what was observed in RIE-1 cells, in both COLO 205 and Caco-2 cells, TRKB^T695I^ and TRKB^D751N^ were less abundantly phosphorylated than wild-type TRKB upon stimulation with BDNF (Figure 6). ERK was phosphorylated upon TRKB stimulation only in Caco-2 cells, but not in COLO 205 cells (Figure 6C,D). This is probably because COLO 205 cells harbor a BRAF^V600E^ mutation (www.sanger.ac.uk/genetics/CGP/cosmic), which is constitutively active and stimulates MAPK signaling [16]. Upon stimulation of Caco-2 cells with BDNF, neither TRKB^T695I^ nor TRKB^D751N^ induced ERK phosphorylation (Figure 6D). Together, these results show that the TRKB^T695I^ and TRKB^D751N^ mutants are less active not only in rat epithelial cells but also in two human colon carcinoma cell lines.

### Knockdown of endogenous TRKB^L138F^ and TRKB^P507L^ in human tumor cell lines

**Figure 7:**
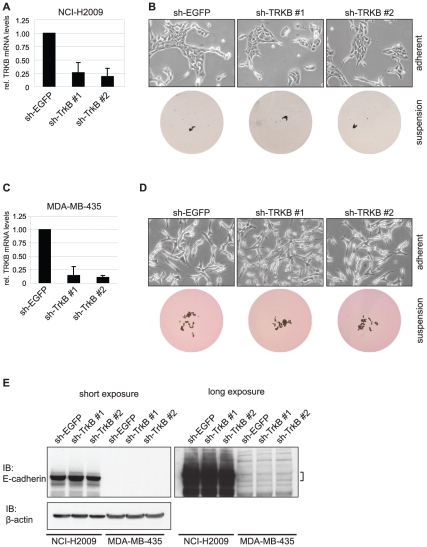
Knockdown of endogenous TRKB^L138F^ and TRKB^P507L^ in tumor cell lines. (**A**) qRT-PCR demonstrating knockdown of TRKB mRNA levels with two non-overlapping short hairpins (sh). Average values of three independent measurements are shown, error bars represent standard deviations. (**B**) Knockdown of TRKB neither affects morphology of adherently growing NCI-H2009 cells, photographed at 50x magnification (top panel), nor anoikis resistance of NCI-H2009 cells in suspension (bottom panel). ULC plates were scanned at 1x magnification 6 days after seeding. (**C**) qRT-PCR demonstrating knockdown of TRKB mRNA levels with two non-overlapping short hairpins. Average values of three independent measurements are shown, error bars represent standard deviations. (**D**) Knockdown of TRKB neither affects morphology of adherently growing MDA-MB-435 cells, photographed at 50x magnification, nor anoikis resistance of MDA-MB-435 cells in suspension (bottom panel). ULC plates were scanned at 1x magnification 6 days after seeding. (**E**) Knockdown of TRKB in NCI-H2009 and MDA-MB-435 cells does not affect E-cadherin protein levels, as assessed by immunoblot (IB) analysis. Long and short exposures of the same blot are shown. β-actin serves as loading control.

The fact that TRKB^L138F^ and TRKB^P507L^ are endogenously expressed in tumor cell lines gave us the possibility to investigate whether they are required for their oncogenic phenotype. We depleted TRKB^L138F^ and TRKB^P507L^ from NCI-H2009 and MDA-MB-435 cells, respectively, by RNA interference with two independent, non-overlapping short hairpin (sh) RNA constructs. We achieved ∼75% downregulation of TRKB in NCI-H2009 cells (Figure 7A). However, this affected neither cell morphology (Figure 7B top panel) nor anoikis resistance (Figure 7B bottom panel). Likewise, ∼80% downregulation of TRKB in MDA-MB-435 cells (Figure 7C) had no effect on cell morphology (Figure 7D upper panel) and anoikis resistance (Figure 7D bottom panel). Consistent with these observations, E-cadherin levels were not changed upon TRKB knockdown in either cell line (Figure 7E). Furthermore, no effect on cell proliferation was observed (data not shown). Notwithstanding that TRKB was not fully depleted in either cell line, these results suggest that (mutant) TRKB in NCI-H2009 and MDA-MB-435 cells is not a major driver of their transformed phenotype.

## Discussion

Three somatic, non-synonymous point mutations of TRKB have been reported in the early large-scale sequencing studies in human cancers: two in colorectal tumors, TRKB^T695I^ and TRKB^D751N^ [17], and one in a lung adenocarcinoma cell line, TRKB^L138F^ [19]. We identified one additional TRKB point mutation, TRKB^P507L^, in the MDA-MB-435 human melanoma cell line. Although some of these mutants have been proposed as candidate cancer-driving genes [17], unexpectedly, our analysis in rat epithelial cells and in human tumor cell lines failed to provide support for a gain-of-function effect for any of the four TRKB point mutations. In fact, the two colorectal tumor-derived TRKB point mutations were associated with even reduced kinase activity and oncogenic potential, compared to wild-type TRKB. As a cautionary note, one has to bear in mind that we did not test the colorectal tumor-derived TRKB mutants in their original context, namely the tumors that endogenously express TRKB^T695I^ or TRKB^D751N^, while cell lines derived thereof are unavailable. Nonetheless, exposure to BDNF of both rat epithelial and human colon cancer cells expressing these mutants resulted in reduced receptor activation of TRKB^T695I^ and almost no activation of TRKB^D751N^. This strongly suggests that these mutants have impaired enzymatic activity in the context of BDNF stimulation. It even raises the possibility that TRKB^T695I^ and TRKB^D751N^ may act in a dominant negative fashion, and that wild type TRKB may also have tumor suppressing activity (or a dual function) in colon cancer, but further studies will be required to address these important issues. We cannot exclude the possibility that the responsiveness of TRKB^T695I^ and TRKB^D751N^ to other TRKB ligands may be different from that to BDNF. We can also not formally exclude the possibility that TRKB^T695I^ and TRKB^D751N^ might have been selected for in the original tumors, contributing to tumor formation with functions that are different from those we tested *in vitro*. These issues not withstanding, we feel it is fair to say that these mutations do not meet conventional criteria for mutational activation of receptor kinases.

Theoretically, all mutations residing in the cytoplasmic part of the receptor could influence the binding of adaptor proteins or substrates of TRKB, independent of the effect on kinase activity. The presence and function of these binding partners would depend on cellular context and may be different in different cell lines. Although the prototypic mechanism for oncogenic function of tyrosine kinases is constitutive or enhanced kinase activity with altered downstream signaling [37], receptor tyrosine kinases can have also kinase-independent functions that contribute to cancer. This has been shown for wild-type EGFR, which, independently of its kinase activity, interacts with and thereby stabilizes sodium/glucose cotransporter 1 (SLGT1) [38]. Downregulation, but not enzymatic inhibition, of EGFR leads to reduced SLGT1 levels, reduced glucose uptake and autophagy of tumor cells [38]. Whether a similar function is also associated with TRKB is not known. A recent study suggests that the kinase-deficient TRKB-T1 splice variant, upon artificial overexpression, may stimulate metastasis of pancreatic adenocarcinoma cells [39]. More research is required to elucidate kinase-independent functions of TRKB and their contribution to malignant disease, in particular to address any role of the endogenous protein in cancer cells.

We have identified and characterized also a novel TRKB point mutation, TRKB^P507L^, which we isolated from the MDA-MB-435 tumor cell line. However, as a normal reference tissue sample derived from the same patient is unavailable, it is unclear whether this mutation corresponds to a somatic mutation in the tumor, to a single nucleotide polymorphism (SNP) from this patient, or was acquired during *in-vitro* passaging. However, this sequence variation has not been reported in public SNP databases.

The ‘Cancer Gene Census’ database (http://www.sanger.ac.uk/genetics/CGP/Census/) is a catalogue of genes whose mutations have been causally implicated in cancer [2]. To date (December 2010), it comprises some ∼436 different genes, amongst which are the TRK receptor family members TRKA (NTRK1) and TRKC (NTRK3), but not TRKB. TRKA and TRKC fusion oncoproteins with constitutive kinase activity have been identified in papillary thyroid (TRKA) [40] and in secretory breast [41] and congenital fibrosarcoma tumors (TRKC) [42]. To date, no such structural mutations for TRKB have been reported in human cancers. Nonetheless, as TRKB is overexpressed in several human malignancies, including neuroblastoma, pancreatic and prostate adenocarcinoma, it has been suggested as a potential target for anticancer therapy [43], [44]. As our analysis presented here fails to show a gain of function effect for four cancer-derived TRKB point mutations, further research will be required before TRKB can be added to the list of *bona fide* cancer genes. If the TRKB point mutants analyzed in this study indeed fail to display gain-of-function transforming properties, this would suggest that they represent passenger mutations and did not drive tumor formation. This may also be indicated by the rare occurrence of these mutations, which, to our knowledge, have not been reported again after their original identification. More recently, four more TRKB point mutations have been identified in human Large Cell NeuroEndocrine Carcinomas (LCNEC) of the lung [45] and another six in lung adenocarcinomas [46]. Although all four LCNEC-derived mutations and four out of the six adenocarcinoma-derived mutations locate to the kinase domain of TRKB, the effect of these novel mutations on gene function remains to be determined.

Our analysis presented here underlines the notion that, in parallel to the ongoing efforts for systematic, large-scale cancer genome sequencing, there is a need for functional studies assessing the biological consequences, particularly the oncogenic potential, of newly discovered cancer-associated mutations. Similar conclusions have been reached by others, for example, on the receptor kinase FLT3 [47]. Our results suggest that the acquisition of point mutations may not correspond to a primary mechanism of TRKB activation in human cancer.

## Materials and Methods

### Sequencing and vector constructs

Genomic DNA for partial sequencing of the *TRKB* gene was isolated by first lysing cells overnight at 55°C in 0.1 M TRIS pH 8.5+0.2 M NaCl +5 mM EDTA +0.2% SDS + 100 µg/ml ProtK, subsequently purifying gDNA with phenol/chloroform/isoamylalcohol and precipitation in isopropanol. Finally, gDNA was washed with 70% ethanol and dissolved in TE. PCR primers used were for LRM domain: exon 7 forward: GTGAAAGAGAGAGAGATCTGG-3′, exon 7 reverse: TGGTATAAAAATAGATCTGC-3′, for kinase domain: exon 16 forward: 5′TGGGGAGTGAGTGCTAACTGG-3′, exon 16 reverse: 5′-CGCCAGTCATCCCTATCAGG-3′, exon 17 forward: 5′-GCCATTTGGGGTGGACTG-3′, exon 17 reverse: 5′-GGATGTGCCCCAAATGTCC-3′, exon 18 forward: 5′-CTCAGTATCATAGGGCCCAC-3′, exon 18 reverse: 5′-GTCCCTTGTTCCCTCCCATG-3′, exon 19 forward: 5′-CCAGCAGCTACAGGGTGGGGG-3′, exon 19 reverse: 5′-CCAGCCTCCAGAGCCATGAG-3′, exon 20 forward: 5′-GTGTCCCCCAGCAGCTCCC-3′, exon 20 reverse: 5′-CCTGACATGGTCTTCCAACCC-3′. PCR fragments were purified on agarose gels and 3-12 ng of DNA was subjected to sequencing analysis using the same primers as mentioned above. Sequences were analyzed with 4peaks software (http://mekentosj.com/4peaks/).

Cloning and expression of human TRKB (NM_006180.3), TRKB^K588M^, TPR-TRKB, and human BDNF (NM_170735) have been described before [25]. All cancer-derived TRKB point mutants were generated by site-directed mutagenesis (Stratagene) according to the manufacturer's instructions. Primers used were for L138F: 5′-CATTTCCGTCAC**T**TTGACTTGTCTGAAC-3′ for P507N: 5′-CCAATGGGAGTAACACTC**T**ATCTTCTTCGGAAGGTGGCCC-3′, for T695I: 5′-GCACCGCGATTTGGCCA**T**CAGGAACTGCCTGGTCGGGG-3′, for D751N: 5′-GGAAATTCACGACGGAAAGC**A**ACGTCTGGAGCCTGGGG-3′ and the respective complementary primers (the bold letter is indicating base substitution C to T for L138F, P705L and T695I, and G to A for D751N). All TRKB mutants and BDNF were expressed from the retroviral pBabe-puro (pBP) and pBabe-hygro (pBH) vectors. Lentiviral short hairpin- (sh-) RNA constructs were generated by cutting pRetroSuper-puro (pRS) [48] sh-EGFP (GCTGACCCTGAAGTTCATC), pRS sh-TRKB#1 (GTAACCTGGTTTCCAAACA) and pRS sh-TRKB#2 (GTAATGCTGTTTCTGCTTA) with NsiI/klenow and XhoI and ligating the respective inserts into an EcoRI/klenow and XhoI–digested HIV-CSCG-EGFP vector, removing EGFP from HIV-CSCG and inserting H1-promotor/shRNA/puromycin-resistance. The sequences in brackets correspond to the targeting sequences of the respective shRNAs.

### Cell culture, retroviral transduction and anoikis assays

RIE-1 cells (a kind gift from R.D. Beauchamp and K.D. Brown [49]), RK3E cells (ATCC), COLO 205 cells (ATCC), Caco-2 cells (ATCC) and MDA-MB-435 cells (ATCC) were cultured in DMEM medium (Gibco) supplemented with 9% fetal calf serum (Greiner bio-one) and penicillin+streptomycin (Gibco). NCI-H2009 cells (a kind gift from F. Kaye [50]) were cultured in RPMI 1640 medium with the same supplements as described above. COLO 205 and Caco-2 cells were transduced with pLZRS-Ecotropic Receptor-IRES-Neo by amphotropic retrovirus produced in BING cells. Retroviral transduction and generation of stable cell pools was done as previously described [25], http://www.stanford.edu/group/nolan/retroviral_systems/phx.html). NCI-H2009 and MDA-MB-435 cells were transduced with lentivirus produced in HEK293 cells.

To induce anoikis we seeded 4*10^5^ freshly trypsinized cells into non-adhesive Ultra Low Cluster (ULC) six-well cell culture dishes (Costar). ULC plates were scanned on an Epson Perfection 4990 Photo scanner and photographs were taken with a Sony DSC-75 digital camera.

### Immunoblotting, immunoprecipitation and antibodies

Cells were lysed in RIPA buffer and analyzed on immunoblots as described before [26]. Antibodies used were: TRK (C-14, Santa Cruz), TRKB (80E3, Cell Signaling), TRKB [TK-] (C-13, Santa Cruz), BDNF (N20, Santa Cruz), E-cadherin (BD Bioscience), phospho-tyrosine (4G10, Upstate), phospho-TRK (phospho-TrkA pTyr^490^, Sigma), phospho-AKT (pSer^473^, Cell Signaling), AKT1/2 (H-136, Santa Cruz), phospho-ERK (phospho p44/42 MAPK pThr^202^/pTyr^204^, Cell Signaling), ERK (p44/42 MAPK, Cell Signaling), β-actin (AC74, Sigma) and α-tubulin (DM 1A, Sigma). Antibodies were diluted 1∶1000-1∶5000 in blocking solution, either 4% Protifar plus (Nutricia) or 4% bovine serum albumin + 1/50 Western Blocking Reagent (Roche). Where indicated, signal intensities were quantified with ImageJ 1.44j software (http://imagej.nih.gov/ij).

For immunoprecipitation, equal amounts of lysates were incubated with 0.5 µg TRK (or TRKB [TK-] as a control) antibody for 2 h at 4°C. Proteins were immobilized with Protein A sepharose beads, washed four times with lysis buffer and immunoblotted for analysis.

For cell surface protein biotinylation assay, cells were washed three times for 10 minutes with ice-cold PBS (pH 8.0) and subsequently incubated for 25 minutes at 4°C with 2 mg/ml freshly prepared Sulfo-NHS-LC-Biotin (Pierce) in PBS pH 8.0. We then quenched excess biotin reagent with PBS + 100 mM glycine by rinsing twice and incubating the cells for 20 minutes at 4°C. Subsequently, cells were lysed, processed for immunoprecipitation and analyzed by immunoblotting as described above. Biotinylated proteins were detected with StreptABComplex/HRP (Dako Cytomation).

### Stimulation with BDNF

Serum-starved cells were stimulated for 5 minutes with serum-free DMEM supplemented with recombinant human BDNF (Peprotech). To inhibit PI3K and MEK activity, cells were pre-treated with 20 µM LY294002 (Calbiochem) and 10 µM U0126 (Cell Signaling) for 30 minutes and subsequently stimulated with recombinant human BDNF in the presence of LY294002 and U0126.

### In vivo assays

Age-matched groups of female Balb/c nude mice were subcutaneously injected with 1*10^4^, 1*10^5^ or 1*10^6^ cells into both flanks. Whereas experiments were generally carried out with pBP-TRKB (and co-expressed pBH-BDNF where indicated), pBH-TRKB^L138F^, pBH-TRKB^wt^ and pBP-BDNF were used for in-vivo assays, shown in independent figure panels. Mice were inspected twice a week and euthanized by CO_2_ when the total tumor burden reached 2 cm^3^ or when tumors started to ulcerate. Tumor size was measured with a caliper and tumor volume calculated by the formula (a*b^2^)/2, with a being the longest diameter and b the perpendicular diameter of the tumor. Kaplan-Meier survival curves were plotted with SPSS 15.0 and statistical significance was determined with Log-Rank tests.

This study and all the procedures applied were approved by the Institutional Animal Experiment Ethics Committee (“Dierenexperimentencommissie” NKI: DEC-nr. 04089), and all efforts were made to minimize suffering of the animals.

### Quantitative reverse transcriptase PCR (qRT-PCR)

Total RNA was isolated using Trizol (Invitrogen) and treated with DNase for 1 h at 37°C (Promega). cDNA was prepared using the reverse transcriptase kit from Invitrogen. Primers were designed with Primer Express software: TRKB-forward: 5′-AACAGAAGTAATGAAATCCCTTCCA-3′, TRKB-reverse: 5′-CAGCATAGACCGAGAGATGTTCC-3′, RPL13 forward: 5′-GAGACAGTTCTGCTGAAGAACTGAA-3′, RPL13 reverse: 5′-TCCGGACGGGCATGAC-3′. Detection was done with SYBR green master mix (Applied Biosystems) on an ABI Prism 7000 thermal cycler (Applied Biosystems). RNA levels were normalized against human RPL13.

## Supporting Information

Figure S1 **Transforming potential of human cancer-derived TRKB point mutants in RK3E cells** ***in vitro*** **.** (**A**) RK3E cells expressing wild-type or mutant TRKB analyzed on immunoblot (IB). Tubulin serves as loading control. (**B**) Morphology of RK3E cells expressing wild-type or mutant TRKB, photographed at 50x magnification. (**C**) Anoikis assay. Cells described in (A) were seeded on ULC plates and scanned at 1x magnification 5 days later. (**D**) Cells described in (A) were transduced with BDNF and analyzed on immunoblot (IB). Arrowhead indicates position of BDNF, which was expressed to lower levels in the sample loaded in the first lane. Tubulin serves as loading control. (**E**) Morphologic transformation of RK3E cells co-expressing mutant or wild-type TRKB and BDNF described in (D), photographed at 50x magnification. (**F**) The epithelial protein E-cadherin is downregulated in TRKB-induced, morphologically transformed, cells, as assessed by immunoblot (IB) analysis. β-actin serves as loading control. (**G**) Anoikis suppression by wild-type or mutant TRKB+BDNF in RK3E cells described in (D). ULC plates were scanned at 1x magnification 5 days after seeding.
(TIF)

Figure S2 **Oncogenic potential of human cancer-derived TRKB mutants in RIE-1 and RK3E cells** ***in vivo*** **.** (**A**) Tumor growth curve for mice shown in Figure 5A. T695I- and D751N-expressing cells did not form tumors for at least 100 days. Data points in Figures S2A-D represent mean values of total tumor burden per mouse, error bars depict standard deviation. (**B**) Tumor growth curve for mice shown in Figure 5B. (**C**) Tumor growth curve for mice shown in Figure 5C. BDNF+D751N-expressing cells did not form tumors for at least 100 days. (**D**) Tumor growth curve for mice shown in Figure 5D. (**E**) 1*10^6^ RK3E cells expressing TRKB (wild-type or mutant) were subcutaneously injected into both flanks of nude mice. n = 3 for wt and P507L, n = 4 for T695I and D751N (**F**) 1*10^6^ RK3E cells expressing TRKB (wild-type or mutant) were subcutaneously injected into both flanks of nude mice. n = 4 for both cell lines. (**G**) 1*10^5^ RK3E cells co-expressing BDNF and TRKB (wild-type or mutant) were subcutaneously injected into both flanks of nude mice. n = 3 for each cell line. Statistical significance was determined with a Log-Rank test. (**H**) 1*10^5^ RK3E cells co-expressing BDNF and TRKB (wild-type or mutant) were subcutaneously injected into both flanks of nude mice. n = 4 for both cell lines. In all experiments, mice were sacrificed when tumor burden reached 2 cm^2^. Kaplan-Meier survival curve is shown. Different litters of mice were used for the different experiments, possibly contributing to experimental variation between experiments.
(TIF)
