# Supplementary figures and images for: Functional Characterization of Human Cancer-Derived TRKB Mutations
Source: PLoS One. 2011 Feb 17;6(2):e16871. doi: 10.1371/journal.pone.0016871 (PMC3040757; doi:10.1371/journal.pone.0016871)

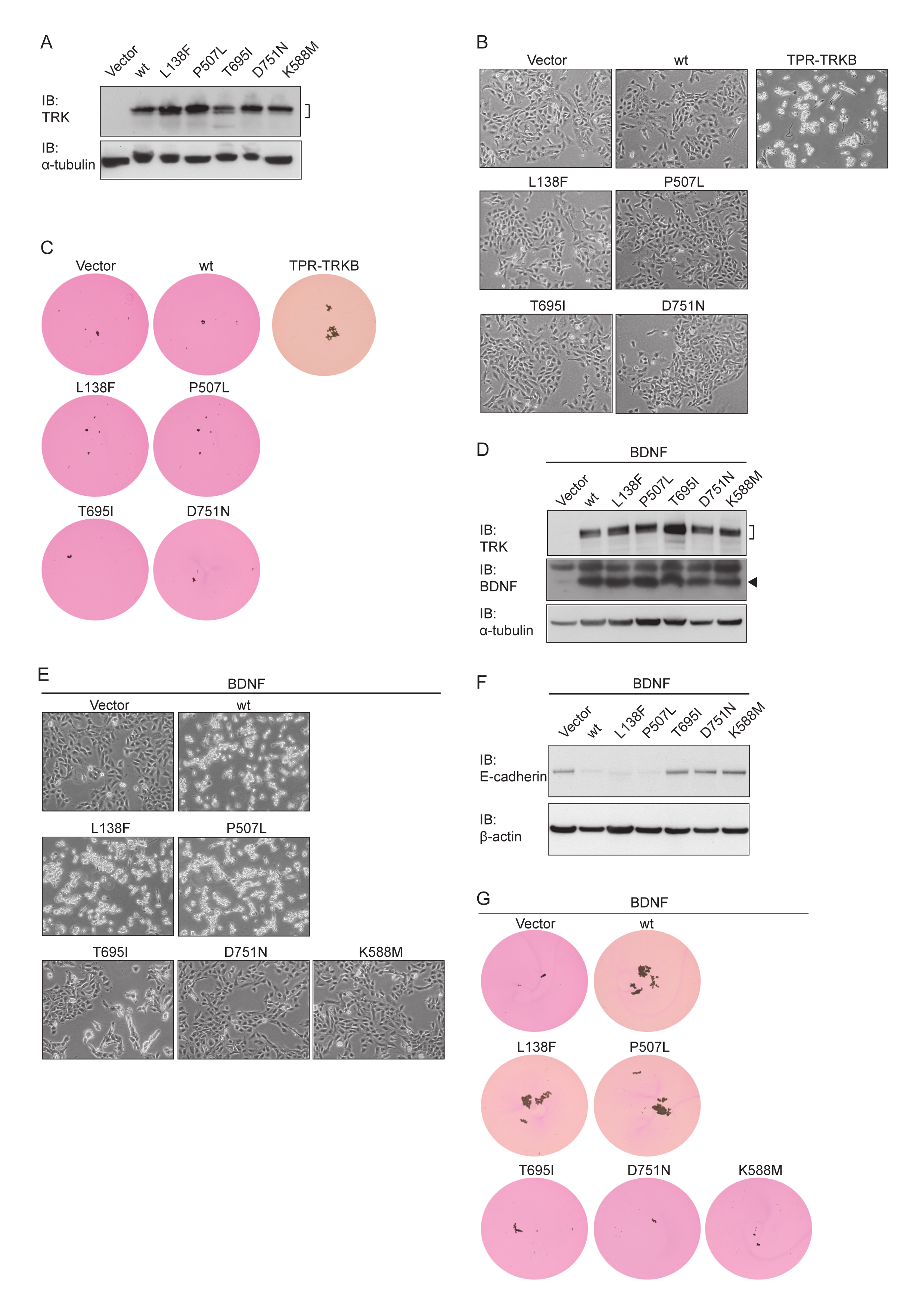

Supplement: Figure S1 — Transforming potential of human cancer-derived TRKB point mutants in RK3E cells in vitro . (A) RK3E cells expressing wild-type or mutant TRKB analyzed on immunoblot (IB). Tubulin serves as loading control. (B) Morphology of RK3E cells expressing wild-type or mutant TRKB, photographed at 50x magnification. (C) Anoikis assay. Cells described in (A) were seeded on ULC plates and scanned at 1x magnification 5 days later. (D) Cells described in (A) were transduced with BDNF and analyzed on immunoblot (IB). Arrowhead indicates position of BDNF, which was expressed to lower levels in the sample loaded in the first lane. Tubulin serves as loading control. (E) Morphologic transformation of RK3E cells co-expressing mutant or wild-type TRKB and BDNF described in (D), photographed at 50x magnification. (F) The epithelial protein E-cadherin is downregulated in TRKB-induced, morphologically transformed, cells, as assessed by immunoblot (IB) analysis. β-actin serves as loading control. (G) Anoikis suppression by wild-type or mutant TRKB+BDNF in RK3E cells described in (D). ULC plates were scanned at 1x magnification 5 days after seeding. (TIF) [file pone.0016871.s001.tif]

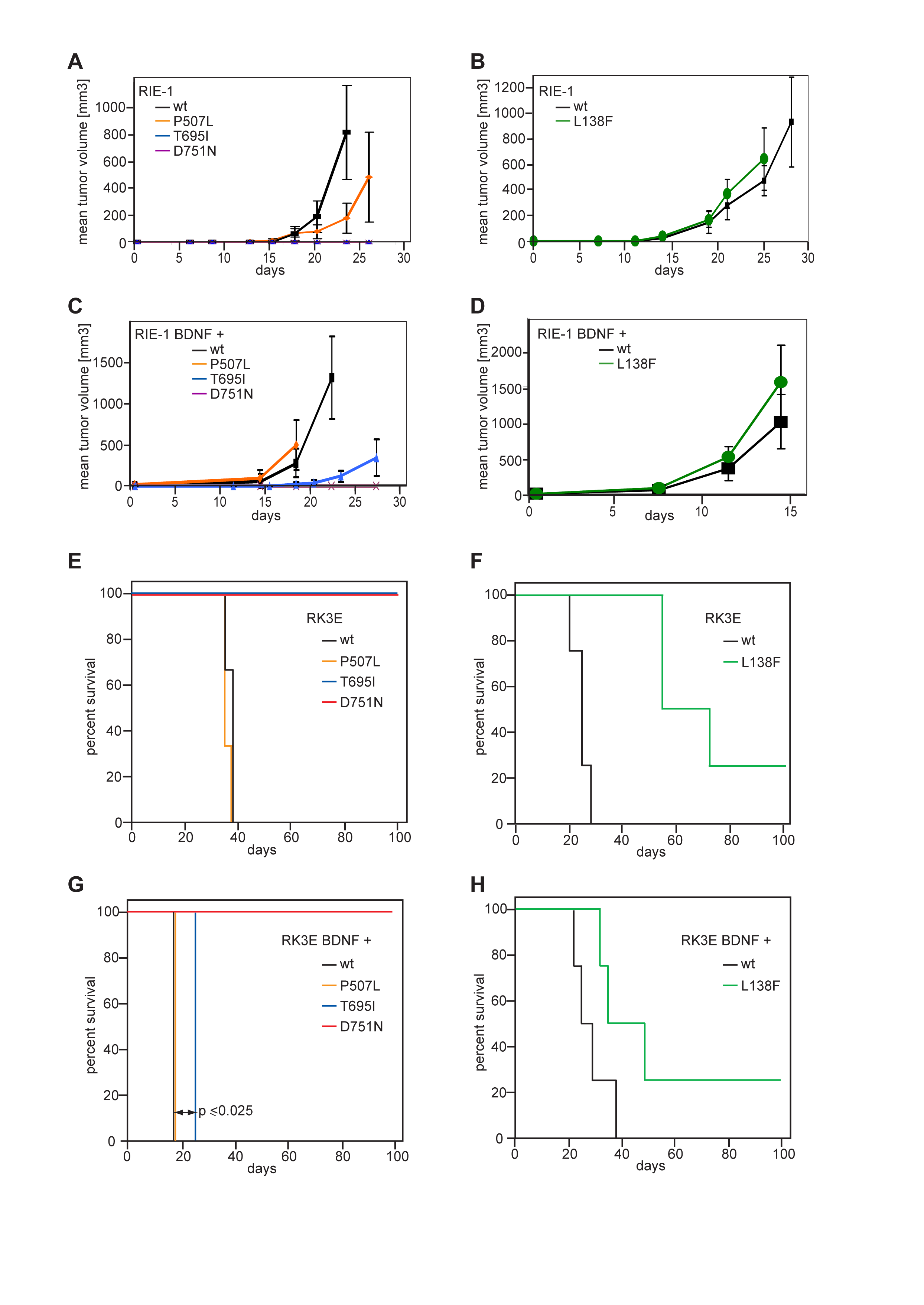

Supplement: Figure S2 — Oncogenic potential of human cancer-derived TRKB mutants in RIE-1 and RK3E cells in vivo . (A) Tumor growth curve for mice shown in Figure 5A. T695I- and D751N-expressing cells did not form tumors for at least 100 days. Data points in Figures S2A-D represent mean values of total tumor burden per mouse, error bars depict standard deviation. (B) Tumor growth curve for mice shown in Figure 5B. (C) Tumor growth curve for mice shown in Figure 5C. BDNF+D751N-expressing cells did not form tumors for at least 100 days. (D) Tumor growth curve for mice shown in Figure 5D. (E) 1*106 RK3E cells expressing TRKB (wild-type or mutant) were subcutaneously injected into both flanks of nude mice. n = 3 for wt and P507L, n = 4 for T695I and D751N (F) 1*106 RK3E cells expressing TRKB (wild-type or mutant) were subcutaneously injected into both flanks of nude mice. n = 4 for both cell lines. (G) 1*105 RK3E cells co-expressing BDNF and TRKB (wild-type or mutant) were subcutaneously injected into both flanks of nude mice. n = 3 for each cell line. Statistical significance was determined with a Log-Rank test. (H) 1*105 RK3E cells co-expressing BDNF and TRKB (wild-type or mutant) were subcutaneously injected into both flanks of nude mice. n = 4 for both cell lines. In all experiments, mice were sacrificed when tumor burden reached 2 cm2. Kaplan-Meier survival curve is shown. Different litters of mice were used for the different experiments, possibly contributing to experimental variation between experiments. (TIF) [file pone.0016871.s002.tif]
